# Implementation of the NutriMental Screener in Psychosomatic Rehabilitation: Findings from Germany and Outlook for National and International Scale-Up

**DOI:** 10.1192/j.eurpsy.2026.11704

**Published:** 2026-07-31

**Authors:** R. Hiltensperger, J. Neher, L. Böhm, J. Kilian, A. S. Müller-Stierlin

**Affiliations:** Institute of Epidemiology and Medical Biometry, Ulm University, Ulm, Germany

## Abstract

**Introduction:**

People with mental disorders frequently experience nutrition-related problems that contribute to somatic comorbidities, reduced occupational functioning, and shortened life expectancy. Yet malnutrition screening tools rarely capture the full range of nutrition risks.

**Objectives:**

The *NutriMental Screener (NMS)* was developed to identify undernutrition, overnutrition, and other nutrition-related problems among patients in mental health settings.

**Methods:**

Following qualitative interviews with service users, the NMS was developed in an international, participatory approach, through stakeholder workshops and two online surveys. The NMS comprises 10 items and can be administered as self-report or interview at admission. A mixed-methods implementation study was conducted in six German rehabilitation clinics, guided by the Consolidated Framework for Implementation Research. Between October 2024 and March 2025, 143 patients and 30 healthcare professionals participated.

**Results:**

Preliminary findings indicate a high number of unmet needs for nutrition-related support among patients with depression in psychosomatic rehabilitation, which are only partially addressed by existing services. Many professionals considered the *NMS* a practical tool to detect nutrition risks early and initiate tailored interventions. At the same time, individual, organizational, and contextual challenges, e.g. limited resources, unclear responsibilities, and institutional routines, were identified as barriers to implementation.

**Conclusions:**

The *NMS* is feasible and acceptable for identifying nutrition risks and guiding patient-centred interventions. To translate evidence into practice, organisational readiness workshops will be conducted in up to eight rehabilitation clinics. Building on the German experience, adaptation and pilot implementation in the UK and Finland are planned to refine implementation strategies and support international uptake.

**Disclosure of Interest:**

None Declared

